# Drug Utilization Study and Safety Pattern of Biologicals in a Tertiary Care Hospital at Eastern India

**DOI:** 10.7759/cureus.108814

**Published:** 2026-05-13

**Authors:** Ravi Roushan, Pranshu Pandit, Saajid Hameed, Nidhi Kumari, Alok Ranjan, Md Jawed Akhtar, Manish Kumar, Hitesh Mishra, Lalit Mohan

**Affiliations:** 1 Department of Pharmacology, Indira Gandhi Institute of Medical Sciences, Patna, IND; 2 Department of Medical Oncology, Indira Gandhi Institute of Medical Sciences, Patna, IND; 3 Department of Anatomy, Indira Gandhi Institute of Medical Sciences, Patna, IND

**Keywords:** adverse events, biologicals, drug utilization, oncology, pharmacovigilance

## Abstract

Background

Biological agents have revolutionized cancer treatment by offering targeted therapeutic effects with improved selectivity over conventional chemotherapy. However, real-world data on their utilization and safety in Indian tertiary care settings remain limited. This study aimed to evaluate the drug utilization pattern and safety profile of biologics in cancer patients.

Methods

A prospective, observational study was conducted over 18 months (July 2022-December 2023) at a tertiary care oncology center in eastern India. A total of 373 prescriptions were analyzed, of which 100 included biological agents and were evaluated in detail. Patients aged 15-70 years with confirmed malignancy were included. Data on demographics, cancer characteristics, drug utilization, and adverse events (AEs) were collected. The primary outcome was the percentage utilization of biologics, while secondary outcomes included comparison of AE incidence between biologics and conventional therapy, and severity assessment using Common Terminology Criteria for Adverse Events (CTCAE) version 5.0.

Results

Biologics were prescribed in 26.8% of total prescriptions. The majority of patients were aged 46-70 years, with a slight female predominance. Breast, lung, and colorectal cancers were the most common malignancies, with most patients presenting at Stage III. Bevacizumab (30/373, 8.04%) was the most frequently used biologic, followed by trastuzumab (27/373, 7.24%) and rituximab (16/373, 4.29%). AE analysis showed that gastrointestinal toxicities such as nausea and vomiting were significantly higher with conventional therapy (61/373, 22.34% vs. 12/100, 12.00%, p=0.03), and neuropathy was also more common (45/273, 16.48% vs. 7/100, 7.00%, p=0.02). In contrast, biologics were associated with significantly higher hematological toxicities, including anemia, neutropenia, thrombocytopenia, and lymphocytopenia (p<0.001), as well as pneumonitis (15/100, 15.00% vs. 11/273, 4.03%, p=0.0008). Most AEs were mild to moderate, with no fatal events reported.

Conclusion

Biologics represent an important component of cancer therapy with an acceptable safety profile. While they reduce gastrointestinal and neurological toxicities, they are associated with higher hematological AEs and pneumonitis, emphasizing the need for careful monitoring.

## Introduction

Cancer remains one of the foremost contributors to global mortality, exerting a profound socio‑economic burden and challenging healthcare systems worldwide [1]. The GLOBOCAN 2020 database projected that “annual cancer incidence would escalate from 19.3 million cases in 2020 to nearly 28.4 million by 2040, representing a striking 47% surge within just five years.” Such projections underscore the urgency of rethinking therapeutic paradigms in oncology [2].

Oncologists increasingly emphasize that conventional cytotoxic chemotherapy has reached the limits of its utility. Its nonspecific mechanism, coupled with cumulative toxicity, renders it less tolerable, particularly in elderly cohorts who constitute the majority of cancer patients [3]. Age‑related comorbidities and diminished physiological reserves exacerbate intolerance to these regimens. Consequently, the spotlight has shifted toward biologics, agents designed to act with molecular precision, sparing normal tissues and thereby mitigating adverse reactions [1-3].

The World Health Organization defines “biologicals” as cellular or gene‑derived therapeutics sourced from humans, animals, or microorganisms, manufactured through biotechnological innovations, and composed of complex macromolecules such as proteins, nucleic acids, or polysaccharides. These may even encompass living entities, cells, and tissues. The category spans vaccines, blood derivatives, somatic cells, recombinant proteins, and gene therapy constructs [4]. Unlike small‑molecule drugs, biologics are inherently heterogeneous, structurally intricate, and sensitive to environmental conditions, necessitating stringent aseptic manufacturing protocols [5,6].

The integration of biologics into precision oncology has been accelerated by next‑generation sequencing, which enables the identification of rare driver mutations in tumor genomes. Tailoring therapy to individual mutational landscapes epitomizes the personalized medicine approach [7].

A holistic perspective on cancer biology recognizes the tumor microenvironment as a critical determinant of disease progression and therapeutic resistance. Stromal cells, immune infiltrates, and extracellular matrix components collectively influence adhesion, migration, proliferation, and drug responsiveness of malignant cells [8-11]. Thus, combinatorial strategies that simultaneously target neoplastic cells and their supportive microenvironment hold promise for superior outcomes [12-14].

Pharmacovigilance of biologics within oncology practice is indispensable [15]. By correlating prescribing trends with adverse drug reaction (ADR) incidence and severity, clinicians can refine therapeutic choices and minimize avoidable harm.

It was hypothesized that the utilization pattern of biologicals could have a significant impact on the incidence of ADRs. Therefore, we planned to investigate whether there would be a significant correlation between the utilization pattern and safety of biologicals. The present study was designed to assess the prescription trends and safety profile of biologicals in oncology. The primary objective was to quantify and compare the percentage utilization of biologic agents among cancer patients. The secondary objectives included analyzing the incidence of adverse events (AEs) in individuals receiving biologics versus those treated with conventional anti‑cancer drugs, and the severity of AEs in accordance with Common Terminology Criteria for Adverse Events (CTCAE) version 5 guidelines.

## Materials and methods

Study overview

This was a hospital-based, prospective, observational study conducted to evaluate the drug utilization pattern and safety profile of biological agents used in cancer patients attending a tertiary care oncology center. The total study duration was 18 months from July 2022 to December 2023, which included patient screening, recruitment, prescription review, follow-up for AEs, data compilation, and statistical analysis.

Study population

The study included patients aged 15-70 years with a confirmed diagnosis of malignancy (ICD‑9/ICD‑10) [16], who were attending the Medical Oncology OPD and receiving biological anti‑cancer therapy. Exclusion criteria comprised individuals with prior organ transplantation, those with rheumatological disorders on immunosuppressive treatment, patients with an expected survival of less than 12 weeks, and women who were pregnant or lactating.

Sample size

The minimum sample size was calculated using the single population proportion formula, assuming a 30% prevalence of biologic utilization [17], with a 95% confidence level (Zα = 1.96) and an absolute precision of 5%. This yielded a requirement of 323 prescriptions. To enhance accuracy and compensate for incomplete data, 373 prescriptions were ultimately analyzed, of which 100 contained biologic agents and were subjected to detailed evaluation of utilization and safety patterns. This sample size calculation was done for the total population (n=373). 

Outcome parameters

The primary outcome of the study was the percentage utilization of biological drugs among total oncology prescriptions, while the secondary outcomes included assessing AE incidence in patients receiving biologics compared to conventional anti‑cancer agents, mapping the distribution of events by organ system, grading severity using CTCAE Version 5.0, and exploring associations between utilization patterns and AE occurrence [18]. Additional analyses focused on demographic trends in ADRs by age and sex, and identification of causative biologics and their pharmacological classes.

Methodology

Data collection was carried out prospectively using a structured case record form, with information obtained from patient prescriptions, OPD treatment charts, laboratory reports, clinical progress notes, and suspected ADR reporting forms. The recorded variables included demographic details (age, sex), cancer diagnosis and stage, drugs prescribed (biological and non‑biological), number and indication of biologics used, AEs observed during therapy, laboratory abnormalities, and the outcome and management of reported reactions. Causality assessment of ADRs was done in the Causality Assessment Meeting held in the Department of Pharmacology (an ADR monitoring center under the Pharmacovigilance Programme of India) in the presence of clinicians and clinical pharmacologists. Unlikely and unclassifiable ADR were excluded. 

For comparative safety assessment, AEs occurring in the biological cohort were compared with those observed in patients receiving conventional anti-cancer drugs. 

Statistical analysis

Data were entered into Microsoft Excel and analyzed using GraphPad version 8.4.3 (GraphPad Software, San Diego, CA). Drug utilization pattern was analyzed as proportions and percentages of total prescriptions. Distribution of malignancies by age, sex, and stage was presented descriptively. Incidence of AEs between biological and conventional therapy groups was compared using the chi-square test or Fisher’s exact test, depending on expected cell counts. p-value <0.05 was considered statistically significant.

## Results

In this observational and prospective study, 373 patients were screened, of whom 100 patients with biologicals in their prescriptions were enrolled. Forty-seven patients in our sample were male, and 53 patients were female. Apart from breast cancer and Hodgkin’s lymphoma, the incidence of other carcinomas was common in male patients.

**Figure 1:**
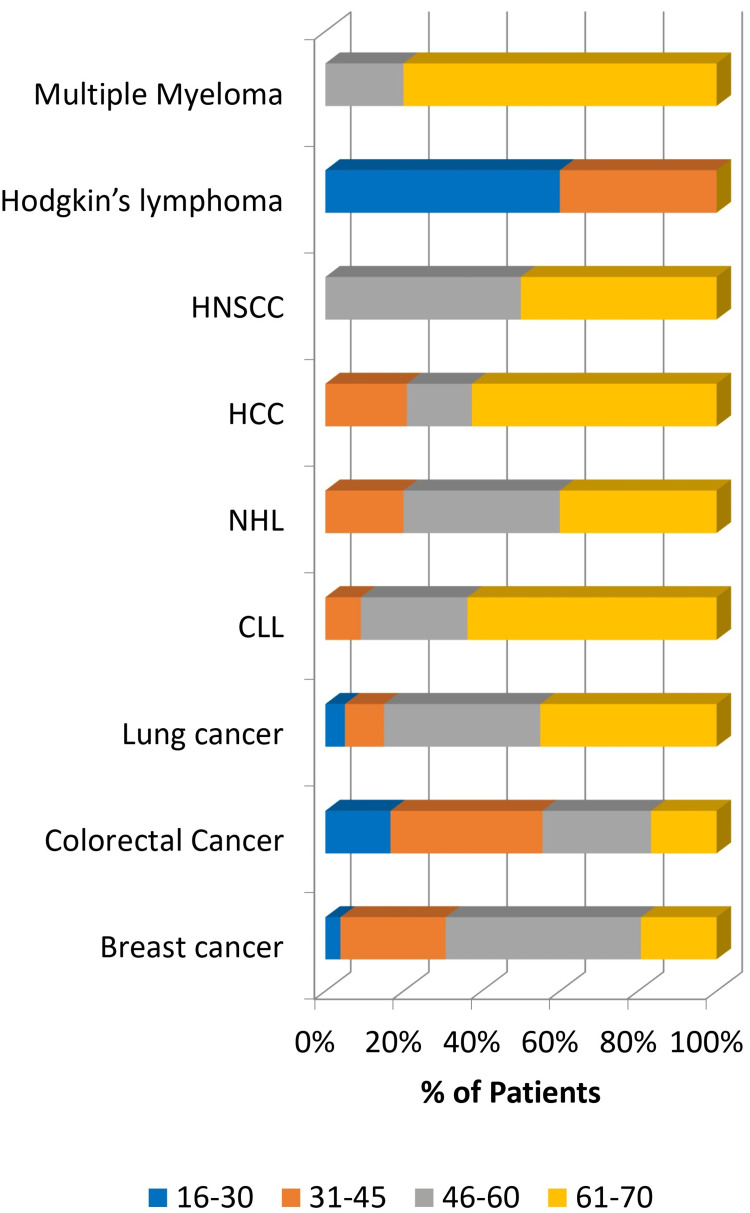
Distribution of Various Neoplastic Disorders With Respect to Age CLL: Chronic Lymphocytic Leukemia; NHL: Non-Hodgkin Lymphoma; HCC: Hepatocellular Carcinoma; HNSCCL: Head and Neck Squamous Cell Carcinoma

Most of the patients were in the 46-60 age group (36%), followed by 61-70 (35%), 31-45 (21%) and 16-30 (8%). Hodgkin’s lymphoma and colorectal cancer were mostly found in younger patients (Figure 1).

Overall, Stage III had the highest proportion of cases (39%), followed by Stage II (28%), Stage IV (19%), and Stage I (14%). Breast cancer was the most common malignancy (26%), with the largest share of patients in Stage III (42.31%), followed by Stage IV (23.08%). Lung cancer accounted for 20% of cases, mainly in Stage III (35%) and Stage II (30%). Colorectal cancer represented 18% of cases, with most patients also presenting in Stage III (38.89%). Among hematological malignancies, CLL constituted 11% of cases, predominantly in Stage II (36.36%), whereas NHL (5%) and Hodgkin’s lymphoma (5%) were mainly seen in Stages II and III. HCC (6%) accouned for half of the cases in Stage III, and HNSCC (4%) was equally distributed between Stages I, II, and predominantly III. Multiple myeloma (5%) was observed mainly in Stages II and III (Table 1).

**Table 1:** Distribution of Neoplastic Disorders Based on Stage (n=100) CLL: Chronic Lymphocytic Leukemia; NHL: Non-Hodgkin Lymphoma; HCC ; Hepatocellular Carcinoma; HNSCCL: Head and Neck Squamous Cell Carcinoma

Neoplastic Disorders	Stage				Total
	I	II	III	IV	
Breast cancer (%)	4 (15.38)	5 (19.23)	11 (42.31)	6 (23.08)	26
Colorectal cancer (%)	3 (16.67)	4 (22.22)	7 (38.89)	4 (22.22)	18
Lung cancer (%)	2 (10.00)	6 (30.00)	7 (35.00)	5 (25.00)	20
CLL (%)	3 (27.27)	4 (36.36)	2 (18.18)	2 (18.18)	11
NHL (%)	0 (0)	2 (40.00)	3 (60.00)	0 (0)	5
HCC (%)	0 (0)	2 (33.33)	3 (50.00)	1 (16.67)	6
HNSCC (%)	1 (25.00)	1 (25.00)	2 (50.00)	0 (0)	4
Hodgkin’s lymphoma (%)	0 (0)	2 (40.00)	2 (40.00)	1 (20.00)	5
Multiple myeloma (%)	1 (20.00)	2 (40.00)	2 (40.00)	0 (0)	5
Total	14	28	39	19	100

Bevacizumab was the most frequently utilized drug, accounting for 8.04% (30/373) of total drug use, mainly prescribed for colorectal cancer, followed by lung cancer and HCC. Trastuzumab was the second most commonly used biological (27/373, 7.24%), predominantly indicated for breast cancer, with additional use in lung cancer. Rituximab constituted 16/373 (4.29%) of total utilization and was mainly used in hematological malignancies such as CLL and NHL. Nivolumab represented 13/ 373 (3.49%) of prescriptions, showing broader indications across lung cancer, HNSCC, HCC, and Hodgkin’s lymphoma. Bortezomib (5/373, 1.34%) was exclusively used for multiple myeloma, while ado-trastuzumab (4/373, 1.07%) was prescribed only in breast cancer cases. Pembrolizumab (3/373, 0.80%) was mainly utilized in HNSCC with limited use in lung cancer, and pertuzumab had the lowest utilization (2/373, 0.54%), restricted to lung cancer (Table 2).

**Table 2:** Utilization of Various Biologicals CLL: Chronic Lymphocytic Leukemia; NHL: Non-Hodgkin Lymphoma; HCC: Hepatocellular Carcinoma; HNSCCL: Head and Neck Squamous Cell Carcinoma

Name of Drug	No of Drugs Utilized	% of Drug Utilized (n=373)	Indication (Number of Drugs Prescribed in That Indication (Stage-n))
Bevacizumab	30	8.04	Colorectal cancer (18 (I-3, II-5, III-11, IV-6)], Lung cancer [9 (I-2, II-3, III-3, IV-2)], HCC [3 (II-1, III-1, IV-1))
Rituximab	16	4.29	CLL (11 (I-3, II-4, III-2, IV-2)], NHL (5 (II-2, III-3))
Trastuzumab	27	7.24	Breast cancer (22 (I-4, II-4, III-9, IV-5)), Lung cancer (5 (II-2, III-2, IV-1))
Nivolumab	13	3.49	Lung cancer (3 (II-1, III-1, IV-1)), HNSCC (2 (I-1, II-1)), HCC (3 (II-1, III-2)), Hodgkin’s lymphoma (5 (II-2, III-2, IV-1))
Bortezomib	5	1.34	Multiple myeloma (5 (I-1, II-2, III-2))
Pertuzumab	2	0.54	Lung cancer (2 (III-1, IV-1))
Ado-trastuzumab	4	1.07	Breast cancer (4 (II-1, III-2, IV-1))
Pembrolizumab	3	0.80	Lung cancer (1), HNSCC (2 (III-2))

Gastrointestinal AEs were generally more common with conventional drugs, with nausea/vomiting showing a statistically significantly higher frequency in conventional therapy (61/273, 22.34% vs. 12/100, 12.00%, p=0.03). Dermatological events such as rash and alopecia were also more frequent with conventional drugs, although the differences were not statistically significant. In contrast, hematological toxicities were significantly higher with biologicals, including. Neuropathy was significantly more common with conventional drugs (45/273, 16.48% vs. 7/100, 7.00%, p=0.02), while pneumonitis occurred significantly more often with biologicals (15/ 100, 15.00% vs. 11/273, 4.03%, p=0.0008). Rare events such as epistaxis, carditis, anaphylaxis, and encephalitis were infrequent in both groups. Meningitis was significantly more frequent in the conventional group as compared to the biologicals (8/273, 2.73% vs 2/100, 2%, p=0.02) (Table 3).

**Table 3:** Comparison of Frequency of Adverse Events between Biologicals and Conventional Drugs

Adverse Events	Number of AE (per 100 Prescriptions)		p-Value (Fisher’s Exact Test)
	Conventional Drugs (n = 273)	Biologicals (n = 100)	
Nausea/vomiting	61 (22.34)	12 (12.00)	0.03
Diarrhea	31 (11.36)	8 (8.00)	0.44
Constipation	9 (3.3)	2 (2.00)	0.73
Abdominal pain	25 (9.16)	6 (6.00)	0.40
Gastritis	16 (5.86)	5 (5.00)	>0.99
Anorexia	32 (11.72)	9 (9.00)	0.58
Pruritus	13 (4.76)	5 (5.00)	>0.99
Rash	52 (19.05)	13 (13.00)	0.21
Alopecia	34 (12.45)	11 (11.00)	>0.99
Lymphocytopenia	16 (5.86)	19 (19.00)	0.0004
Neutropenia	7 (2.56)	15 (15.00)	<0.0001
Anemia	20 (7.33)	34 (34.00)	<0.0001
Thrombocytopenia	14 (5.13)	21 (21.00)	<0.0001
Impaired liver function	13 (4.76)	6 (6.00)	0.60
Myositis	19 (6.96)	3 (3.00)	0.21
Neuropathy	45 (16.48)	7 (7.00)	0.02
Pedal edema	4 (1.47)	2 (2.00)	0.66
Epistaxis	0 (0)	2 (2.00)	0.07
Meningitis	8 (2.73)	2 (2.00)	0.02
Encephalitis	5 (1.83)	1 (1.00)	>0.99
Carditis	0 (0)	1 (1.00)	0.26
Anaphylaxis	0 (0)	2 (2.00)	0.07
Pneumonitis	11 (4.03)	15 (15.00)	0.0008

**Figure 2:**
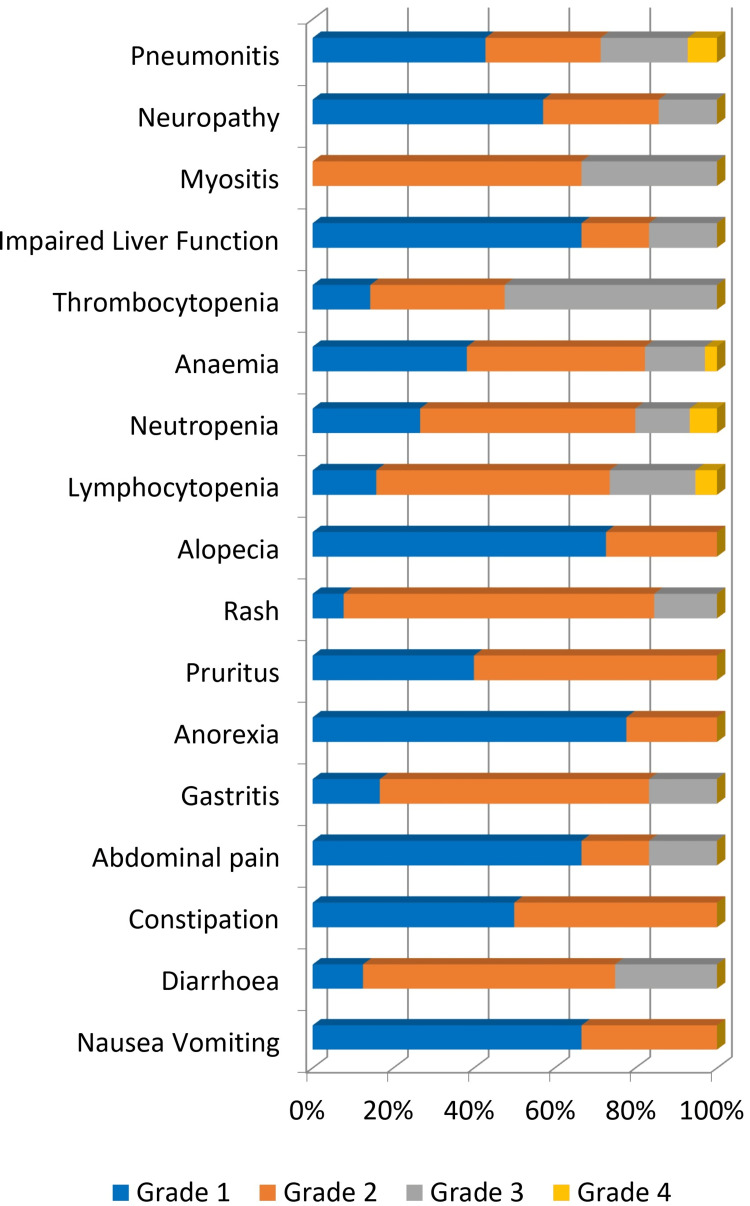
Distribution of Adverse Events with Respect to CTCAE Grade CTCAE: Common Terminology Criteria for Adverse Events

Most of the gastrointestinal adverse effects belonged to grade 1 or 2 CTCAE. A significant proportion of hematological adverse effects belonged to grade 3 severity as per CTCAE. Grade 4 CTCAE AEs were two cases of anaphylaxis, and one case each of lymphocytopenia, neutropenia, anemia, encephalitis, and pneumonitis. There was no case of grade 5 CTC AE (Figure 2).

## Discussion

The present study was undertaken to evaluate the drug utilization pattern and safety profile of biological agents among cancer patients attending a tertiary care oncology center in eastern India. Our findings demonstrate that biologics constituted a modest but clinically significant proportion of total oncology prescriptions, with monoclonal antibodies targeting vascular endothelial growth factor and human epidermal growth factor receptor emerging as the most frequently prescribed agents. The safety analysis revealed a distinct toxicity spectrum, wherein biologics were associated with significantly higher rates of hematological adverse events and pneumonitis, whereas conventional chemotherapeutic agents exhibited greater gastrointestinal and neurological toxicities. Importantly, the majority of adverse events were mild to moderate in severity, with no fatal outcomes recorded during the study period.

The utilization pattern observed in our cohort aligns partially with trends documented in large-scale observational studies from developed healthcare systems, although notable divergences exist. In the present investigation, bevacizumab emerged as the most frequently prescribed biologic, predominantly indicated for colorectal carcinoma, followed by lung cancer and hepatocellular carcinoma. This finding is consistent with that of Hess et al. (2010), who reported bevacizumab as the most commonly administered biologic therapy in metastatic colorectal cancer, with FOLFOX plus bevacizumab being a predominant first-line regimen [19]. Similarly, Abrams et al. (2014) demonstrated that bevacizumab was integrated into treatment protocols in approximately 51% of first-line settings in metastatic colorectal cancer [20]. The concordance between our findings and those of Hess et al. (2010) and Abrams et al. (2014) underscores the global acceptance of anti-angiogenic therapy in advanced malignancies [19,20].

However, the magnitude of biologic utilization in our study was considerably lower than that reported in these Western cohorts. While approximately 27% of prescriptions in our study included biologics, Hess et al. (2010) reported a biologic utilization rate of nearly 69% [19]. However, Hess et al. evaluated this utilization in only colorectal cancer patients as compared to all cancer types in our study. This disparity likely reflects differences in healthcare infrastructure, economic constraints, and access to costly therapies. In the Indian context, particularly in public healthcare settings, out-of-pocket expenditure remains a major barrier to accessing biologic treatments. Limited insurance coverage, variations in reimbursement policies, and delayed incorporation of precision oncology practices further contribute to reduced utilization compared to resource-rich settings.

The pattern of trastuzumab use in our study also aligns with global evidence. Trastuzumab was the second most frequently prescribed biologic and was primarily used in breast cancer patients. This is comparable to findings by Ray et al. (2013), who identified trastuzumab and bevacizumab as the most commonly utilized biologics in metastatic breast cancer across multiple lines of therapy [21]. The similarity between our results and those of Ray et al. (2013) suggests that evidence-based biologic therapies are being appropriately implemented in Indian tertiary care centers when available [21].

Regarding immunotherapy, our study documented modest use of immune checkpoint inhibitors such as nivolumab and pembrolizumab across malignancies, including lung cancer, head and neck cancers, hepatocellular carcinoma, and Hodgkin’s lymphoma. Carroll et al. (2023) reported that most oncology practices in the United States adopted immunotherapy within two years of regulatory approval, although adoption varied by practice size and geographic location [22]. While direct comparison is limited by study design, our findings indicate a gradual but increasing adoption of immunotherapy in the Indian setting. Furthermore, Yuan et al. (2019) emphasized that treatment decisions in non-small-cell lung cancer should be guided by tumor molecular profiling and optimal combinations of targeted therapy, immunotherapy, and chemotherapy [23]. This evolving paradigm appears to be gradually reflected in our clinical practice.

The issue of differential effectiveness of biologics across populations, though not directly examined in our study, is highlighted in existing literature. Goel et al. (2017) reported that biologic therapy improved survival outcomes in metastatic colorectal cancer, but subgroup analysis suggested variation across racial groups [24]. However, Goel et al. (2021) later demonstrated that biologic therapies provided comparable survival benefits across different racial populations [25]. Similarly, Patel et al. (2024) found that biologic therapy significantly improved survival among Hispanic patients with metastatic colorectal cancer. These findings emphasize the importance of equitable access to biologic therapies and caution against overgeneralization of results from homogeneous study populations [26].

The safety profile observed in our study revealed important distinctions between biologic and conventional therapies. A significantly higher incidence of hematological toxicities, including anemia, neutropenia, thrombocytopenia, and lymphocytopenia, was observed in patients receiving biologics. This contrasts with the traditional understanding of chemotherapy-induced myelosuppression and may reflect the influence of combination regimens, prior therapies, or patient-specific factors. Additionally, pneumonitis was significantly more common in the biologic group, consistent with known adverse effects of immune checkpoint inhibitors and certain targeted therapies, necessitating vigilant monitoring.

In contrast, conventional chemotherapeutic agents were associated with higher rates of gastrointestinal AEs, particularly nausea and vomiting, as well as neurological toxicities such as peripheral neuropathy. These findings are consistent with established toxicity profiles and suggest that biologics may offer advantages in reducing certain debilitating side effects, thereby improving patient quality of life.

Age-related considerations further contextualize these findings. Zhang et al. (2015) demonstrated that targeted therapies provided superior progression-free and overall survival compared to single-agent chemotherapy in elderly patients with advanced non-small-cell lung cancer, along with a more favorable toxicity profile [27]. Similarly, Meoni et al. (2013) concluded that targeted therapies are particularly beneficial in elderly patients due to better tolerability [28]. Given that many patients in our cohort were in the fifth to seventh decades of life, the observed toxicity patterns may inform individualized treatment decisions in older populations.

The predominance of advanced-stage presentation (Stage III and IV) in our study reflects a common challenge in the Indian healthcare system: late diagnosis. Nagasaka and Gadgeel (2018) highlighted that while chemotherapy offers modest survival benefits in early-stage disease, targeted therapies and immunotherapies hold promise for improving outcomes across disease stages [29]. In our setting, biologics were primarily used in advanced disease, indicating potential scope for earlier integration as access improves.

Despite its strengths, our study has certain limitations. The single-center design and relatively small sample size limit generalizability. The absence of long-term follow-up data restricts assessment of survival outcomes, and lack of detailed subgroup analysis precludes evaluation of agent-specific effects. Additionally, regional variations in healthcare access across India may influence utilization patterns. Oncologists' preference for conventional drugs or biological drugs could be affected by the type and location of cancer, but this study gives an overall comparison and highlights the need for further comparative studies in a specific type and location of malignancy. 

## Conclusions

The present study demonstrates that biological agents constitute a significant and growing component of anticancer pharmacotherapy, with bevacizumab and trastuzumab emerging as the most frequently prescribed biologics, predominantly utilized for colorectal, breast, and lung malignancies in advanced disease stages. The safety analysis reveals a distinct and clinically relevant toxicity spectrum, characterized by significantly higher rates of hematological AEs including anemia, thrombocytopenia, lymphocytopenia, and neutropenia, as well as an elevated incidence of pneumonitis among biologic recipients, whereas conventional chemotherapeutic agents are associated with greater gastrointestinal and neurological toxicities. Importantly, the majority of AEs were of mild to moderate severity with no fatal outcomes, suggesting that biologic therapies, when administered with appropriate monitoring and supportive care, demonstrate an acceptable safety profile in the Indian tertiary care setting.
